# Enactive Approach and Dual-Tasks for the Treatment of Severe Behavioral and Cognitive Impairment in a Person with Acquired Brain Injury: A Case Study

**DOI:** 10.3389/fpsyg.2016.01712

**Published:** 2016-11-01

**Authors:** David Martínez-Pernía, David Huepe, Daniela Huepe-Artigas, Rut Correia, Sergio García, María Beitia

**Affiliations:** ^1^Center for Social and Cognitive Neuroscience, School of Psychology, Universidad Adolfo IbáñezSantiago, Chile; ^2^Experiential Neurorehabilitation Research Department, Fundación PolibeaMadrid, Spain; ^3^Laboratory of Experimental Psychology and Neuroscience, Institute of Cognitive and Translational Neuroscience, INECO Foundation, Favaloro UniversityBuenos Aires, Argentina; ^4^Faculty of Education, Universidad Diego PortalesSantiago, Chile

**Keywords:** enaction, seated affordance, dual-tasks, neurorehabilitation, behavioral disorder, cognitive impairment

## Abstract

One of the most important sequela in persons who suffer from acquired brain injury is a behavioral disorder. To date, the primary approaches for the rehabilitation of this sequela are Applied Behavior Analysis, Cognitive-Behavior Therapy, and Comprehensive-Holistic Rehabilitation Programs. Despite this theoretical plurality, none of these approaches focuses on rehabilitating behavioral disorders considering the relation between affordance and environmental adaptation. To introduce this therapeutic view to neurorehabilitation, we apply the theoretical tenets of the enactive paradigm to the rehabilitation of a woman with severe behavioral and cognitive impairment. Over seventeen sessions, her behavioral and cognitive performance was assessed in relation to two seated affordances (seated on a chair and seated on a ball 65 cm in diameter) and the environmental adaptation while she was working on various cognitive tasks. These two seated affordances allowed to incorporate the theoretical assumptions of the enactive approach and to know how the behavior and the cognition were modified based on these two postural settings and the environmental adaptation. The findings indicate that the subject exhibited better behavioral (physical and verbal) and cognitive (matching success and complex task) performances when the woman worked on the therapeutic ball than when the woman was on the chair. The enactive paradigm applied in neurorehabilitation introduces a level of treatment that precedes behavior and cognition. This theoretical consideration allowed the discovery of a better relation between a seated affordance and the environmental adaptation for the improvement behavioral and cognitive performance in our case study.

## Introduction

Persons who suffer from acquired brain injury have multiple impairments that prevent their performing activities of daily life, such as locomotion, self-care, communicating, and reasoning, normally. In addition, such persons suffer from mood changes (depression, anxiety). Nevertheless, one of the most important problems of the brain damaged is the behavioral disorder (Lezak, 1986; Tate, 1986; Brooks et al., 1987; Burke and Wesolowski, 1988; Livingston and Brooks, 1988; Jacobs, 1998). The magnitude and persistence of these behavioral sequelae suggest that the problem extends beyond the individual sphere, affecting the person’s social and familiar contexts such as emotional overburdening in a familiar environment (Godfrey et al., 1991; Franulic et al., 2000), problems with community integration and psychosocial adjustment (Milders et al., 2003; McCabe et al., 2007). In addition to all of these sequelae, the behavioral disorder may be a serious obstacle to the rehabilitation process and the patient’s recovery (Sohlberg and Mateer, 2001; Wood and McMillan, 2001; van Reekum et al., 2005).

Behavioral disorders are commonly classified into externalizing (impulsiveness, irritability, aggression, loss of emotional control, hyperactivity) and internalizing (depression, withdrawal, apathy) symptoms in both childhood (Cicchetti and Toth, 2014) and adulthood (John et al., 2008). The persistency of these symptoms is such that they may be present for many years after brain injury (Kelly et al., 2008). The severity of this impairment depends on various factors such as premorbid behavior, personal skills, extent of brain injury, and the types of physical, emotional, and cognitive sequelae the person suffers (Eames et al., 1990; Mateer and Ruff, 1990; Davis and Goldstein, 1994).

To date, the primary approach for the treatment of behavioral disorder following acquired brain injury is based on behavioral therapy (McGlynn, 1990; Jacobs, 1993; Wesolowski and Zencius, 1994). According to the majority of the modern literature (Ylvisaker et al., 2005, 2007; Cattelani et al., 2010; Geurtsen et al., 2010), these interventions may be organized into three main categories: Applied Behavior Analysis, comprising contingency management procedures and positive behavior intervention; Cognitive-Behavior Therapy; and Comprehensive-Holistic Rehabilitation Programs. Each of these approaches has its own theoretical and therapeutic assumptions that target specific features of behavioral disorders.

Despite this theoretical plurality, none of these therapeutic approaches focuses on rehabilitating behavioral disorder considering the relation between affordance and environmental adaptation. Current perspectives do not consider how physical structure and the environment compose the first step in the emergence of behavior and cognition. The theoretical assumptions of conventional behavioral therapies are based on the dichotomy between subject and object. The subject and the object cause a natural division between the person who suffers from the disability and the environment that surrounds that person. For example, Applied Behavior Analysis focuses on manipulating the environment to improve misbehavior. In Cognitive-Behavior Therapy, the intervention is based on improving self-consciousness and learning cognitive strategies. And finally, the third group, the Comprehensive-Holistic Rehabilitation Programs, simultaneously incorporate the manipulation of the environment and cognition to recover from behavioral disorders (Cattelani et al., 2010). Nevertheless, and opposed to these therapeutic models, the enactive view is not based on the division between subject and object. The enactive approach assumes the process of interaction, constant, and unbreakable, between the environment and the sensorimotor schemas for the emergence of behavior and cognition. In the view of the therapeutic approach that we introduce in this publication, it indicates that any clinical intervention must consider the interaction between body and environment to improve the neurological sequelae. Following we briefly explain the enactive approach.

Enaction is a novel paradigm in the cognitive sciences (Di Paolo et al., 2010) that was initially articulated by Varela et al. (1991) in “*The Embodied Mind*.” In this approach, behavior and cognition develop through a dynamic interaction between the physiology of the organism, the sensorimotor systems, and the environment (structural coupling between the body and the world). Human beings enact the world; their embodied actions in the world are the first steps in the development of perception and the basis of cognition. In opposition to other theories of the mind in which the subject and the environment are considered to be separate entities, enaction claims that the study of the action and the cognition requires the simultaneous study of the mind, the body and the environment because all three are indissolubly intertwined in the mind processes (Thompson, 2007). Originally, this paradigm focused primarily on simple cognitive processes such as color perception (Varela et al., 1991). Currently the enactive approach addresses the explanation of action and cognition in activities that require high-level cognitive processes such as mathematics (Núñez, 2010), language (Bottineau, 2010), the human brain (Engel, 2010), social interaction (Di Paolo et al., 2010), and emotion (Colombetti, 2010). However, the enaction approach has not been applied in neurological therapy.

To introduce this therapeutic view to the field of neurorehabilitation, we apply the theoretical tenets of enaction to clinical practice. Thus, to research this interacting system of body structure and the environment, we assessed, during 17 sessions, the behavioral and cognitive performances of a woman with a severe acquired brain injury (ABI) in two different therapeutic contexts. Following Gibson’s (2014) works about ecological perception we call these settings seated *affordances*. In one seated affordance, the woman was required to perform various cognitive tasks in the traditional posture of cognitive rehabilitation, that is, seated on a chair. In the other seated affordance, the woman was required to attempt the same tasks that she had performed in the chair; this time, however, the woman was seated on a therapeutic ball (65 cm in diameter). In both seated affordances, misbehaviours (physical and verbal), and the successes and failures of the cognitive tasks were recorded during the performance of the cognitive tasks.

We formulated a primary hypothesis and a peripheral hypothesis based on prior clinical experiences. The primary hypothesis was that the sensorimotor dynamics between the body and the environment in the ball condition would allow a better modulating effect of the externalizing symptoms than when the woman was seated on a chair. The peripheral hypothesis, considering that enaction claims that motor action is directly linked to cognition, was that the woman achieved a better cognitive performance working in the ball condition than in the chair condition. A multiple schedule design was developed to examine how these bodily and environmental adjustments modified the behavior and cognitive variables.

## Case Report

### The Subject

We applied the experimental design to a 36-year-old woman who, in 2007, suffered a severe brain injury. The injury occurred 48 h after a normal childbirth, at which time the woman suffered from a severe encephalopathy secondary to fulminant hepatic failure. At the emergency service, cerebral oedema and multiple non-specific lesions in bilateral white matter were observed. One week later, the woman was diagnosed with preeclampsia. Afterward, she received a liver transplant. Ultimately, she began her rehabilitation in a specialized center for neurological therapy.

At the time of this research, 6 years after the ABI, the neuropsychological assessment was only qualitative because of severe behavioral and cognitive disorders. During the exploration, the woman was restless, impulsive, uninhibited, and verbally incoherent. Orientation to person place and time was impaired.

Although the neuropsychological impairments were generalized in all cognitive functions, with regard to this study, the most important injuries were associated with attention, reasoning, comprehensive and expressive aphasia, and executive functions.

The subject was highly impaired in sustained, selective, alternant, and divided attention. The woman was incapable of paying attention because of extreme distractibility. Her language was verbose and meaningless, with alterations in grammar, use of neologisms, palilalia, and echolalia. The subject made inappropriate comments and numerous perseverations without being able to maintain social relationships. The woman could understand simple sentences but struggled with the pragmatic elements of language. Her executive functions were severely affected, functions such as planning and sequencing capabilities and mental flexibility. The woman could not focus her attention on relevant stimuli or omit irrelevant stimuli. The woman could not inhibit verbal and motor behaviors because of her impulsivity and made decisions and solved problems without reflection because her abstract and complex reasoning were extremely affected.

Physically, the woman could move and walk without any external support and was capable of organizing her movements adequately, both fine motor and gross motor skills. Therefore, the subject did not have any difficulty in her physical posture or balance.

This study was carried out in accordance with the recommendations of Fundación Polibea’s ethical committee. Family of the person who participated in this study gave written informed consent in accordance with the Declaration of Helsinki.

### Procedures

The experimental design was applied in seventeen sessions. In each session, two cognitive tasks were rated, and different behavioral and cognitive variables were collected while the person was seated on different affordances (on a chair or on a ball). Behavioral and cognitive variables were assessed on both seated affordances; however, to counterbalance, the data from the sessions were assessed changing the order of the affordance (Session 1: First on the ball and second on the chair. Session 2: First on the chair and second on the ball, etc.). Between changing from one seated affordance to the other seated affordance, the task was stopped for 5 min.

When the woman was seated on the chair, we used the standard posture of a traditional cognitive session. The subject had to be seated in a chair with her feet on the floor. Moreover, the subject had to keep her trunk erect without resting against the chair back although she was allowed to place her arms on the table. In the second seated affordance, we exchanged the chair for a therapeutic ball (65 cm in diameter). The woman had to keep her trunk straight with both feet on the floor, neither foot touching the ball to avoid her using her ankles for stabilization. The researcher did not allow the woman to rest her arms on the table.

In both seated affordances, two cognitive exercises were implemented. The first exercise required the woman to perform a matching task, and the second task required the woman to utilize greater cognitive resources (complex task). In the matching exercise, the woman worked on visual perception and sustained attention, and the complex task involved auditory perception, language comprehension, visual perception, selective attention, and motor skills. The performance of the first exercise (the matching task) comprised giving the woman cards, one by one, to match to the card with the identical figure located on the table in front of her. The exercise lasted 15 min, and during the session, the therapist recorded the number of successes and failures. Then, the second exercise (complex task) began. This exercise comprised the researcher’s asking her to point to a specific day of the week on a timetable. This performance was repeated seven times and the number of successes recorded.

During the sessions, there were two therapists in the room. One therapist, the psychologist, managed the psychological session seated in front of the woman on the other side of the table, and the other therapist, the physical therapist, was located behind the woman checking to see whether the woman touched the ball with her ankles.

All sessions were recorded on video for later study to allow various researchers to assess the behavioral variables. The score of disruptive behavior was computed as the number of laughs, grabs, strikes to the therapists, and looks back to the second therapist. Self-verbalization was computed by the number of times the woman talked without communicative intention. Finally, the verbalization variable was computed by the number of times the woman talked to one of the therapists.

### Experimental Design

We applied a multi-treatment design (Kazdin, 1982) and more specifically, a multi-schedule design (Hersen and Barlow, 1978; Kazdin, 1982). The primary feature of this single-case design is that separate interventions are associated with distinct stimulus conditions. This methodological design is consistent with the goal of this research because “after the stimulus has been associated with its respective intervention, a clear discrimination is evident in performance” (Kazdin, 1982, p. 173).

The data analysis began by assessing the autocorrelation of all variables in both the control phase and the experimental phase. The variables that were not autocorrelated were matching failure (on the chair, *r* = -0.18, *p* = 0.51, ns; on the ball, *r* = 0.23, *p* = 0.38, ns.), disruptive behavior (on the chair, *r* = -0.014, *p* = 0.96, ns; on the ball, *r* = -0.26, *p* = 0.33, ns.), verbalization (on the chair, *r* = 0.318, *p* = 0.23, ns; on the ball, *r* = 0.36, *p* = 0.17, ns.), self-verbalization (on the chair, *r* = 0.25, *p* = 0.35, ns; on the ball, *r* = 0.15, *p* = 0.57, ns.), and complex tasks (on the chair, *r* = -0.18, *p* = 0.50, ns; on the ball, *r* = 0.28, *p* = 0.30, ns.). The only variable that showed autocorrelation was matching success and only in the chair condition (*r* = 0.60, *p* < 0.05 on the chair compared with on the ball, *r* = -0.30, *p* = 0.26, ns.). We processed this analysis in various manners depending on whether the variables were autocorrelated. The non-autocorrelated variables were assessed with the Mann–Whitney *U* test. This analysis is considered the most appropriate and strict for this type of data (Tate and Perdices, 2012). Relatively, the autocorrelated variable was assessed with c-statistics according to the proposal suggested by Tryon (1982) and DeCarlo and Tryon (1993). This analysis allows detecting small changes in successive measurements.

Three researchers were employed to increase the internal validity of the observational variables (disruptive behavior, self-verbalization, and verbalization). These researchers conducted the independent measurement. In addition, the intra-class correlation coefficient was analyzed for each variable. The measurements of this analysis showed a high reliability of the intra-class correlation coefficient (ICC Point Estimate [95% CI]): disruptive behavior 0.949 (0.900–0.979), self-verbalization 0.932 (0.865–0.972), and verbalization 0.937 (0.876–0.974).

## Results

We identified significant differences between the performance of the person in the ball condition and in the chair condition, which are summarized in **Table 1**.

**Table 1:** Mann–Whitney *U* test results.

Variables	Seated affordance	Mean (SD)	Sum of ranks	*z*-value	*p*-value
Matching failure	On the chair On the ball	1.76 (1.751) 3.88 (2.421)	217 378	-2,823	0.00^∗∗^
Disruptive behavior	On the chair On the ball	50.41 (24,819) 22.27 (13,929)	400.50 194.50	-3,548	0.00^∗∗^
Verbalization	On the chair On the ball	150.44 (68.846) 86.29 (36.308)	387.00 208.00	-3,083	0.00^∗∗^
Self-verbalization	On the chair On the ball	169.97 (44.165) 192.86 (34.384)	240 355	-1,981	0.02^∗^
Complex task	On the chair On the ball	1.88 (1.054) 2.53 (1.068)	250.50 344.50	-1,740	0.04^∗^

*^∗^Statistically significant (*P* < 0.05). ^∗∗^Statistically significant (*P* < 0.01).*

**FIGURE 1:**
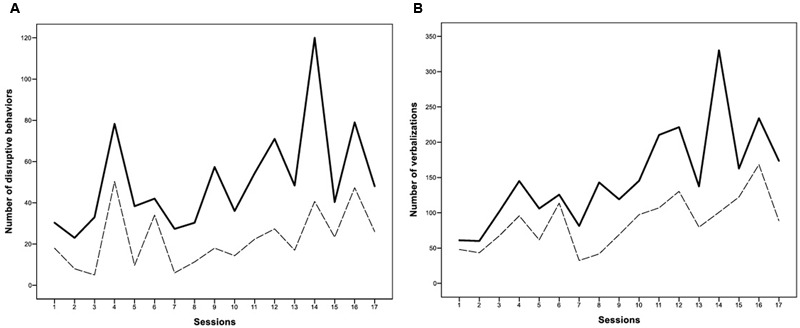
**Both **(A and B)** figures show that variables behaved quite similarly in the sessions; a larger number of misbehaviours or verbal productions were collected during the sessions with both seated affordances.** Nevertheless, one consistent element in all sessions is that behavioral variables that developed in the ball condition (- - -) were always significantly smaller than in the chair condition (-).

The variables assessed with the Mann–Whitney *U* test had significant results. For the behavior variable, a significantly lower number of disruptive behaviors were observed on the ball (**Figure 1A**), *Z*_0_ = -3.55, *p* < 0.001, than on the chair. The sum of the ranks was 400.50 for the chair condition and 194.50 for the ball condition. For the verbalization variable, the woman decreased her communication when working on the ball (**Figure 1B**), *Z*_0_ = -3.08, *p* < 0.001. The sum of the ranks was 387 for the chair condition and 208 for the ball condition.

We observed that the subject accomplished a better complex task performance while working on the ball, *Z*_0_ = -1.74, *p* < 0.05. The sum of the ranks was 250.50 for the chair condition and 344.50 for the ball condition. When we assessed the manner in which the subject developed the task, the psychologist who guided the sessions did not observe that the woman modified any of the strategies used to successfully accomplish the task (visual strategy or a longer time scanning).

By contrast with our initial hypothesis, both the self-verbalization variable and the matching failure variable were significantly higher in the ball condition than in the chair condition. For self-verbalization, the person significantly increased her verbal response when working on the ball, *Z*_0_ = -1. 98, *p* = 0.02. The sum of the ranks was 240 for the chair condition and 355 for the ball condition. In the matching failure, the person made significantly more mistakes working on the ball, *Z*_0_ = -2.82, *p* < 0.001. The sum of the ranks was 217 for the chair condition and 378 for the ball condition.

Finally, the only autocorrelated variable (matching success) was the applied c-statistic. Assessing the baseline, a significant trend was observed: c-statistic = 0.62 (*SE* = 0.22), z (two-tailed) = 2.82, *p* < 0.01 (*p* = 0.0024). Following the analysis protocol, the difference between the treatment line (on the ball) and the baseline (on the chair) for each session (days) was calculated. The results showed a better matching success when working on the ball: c-statistic = 0.90 (*SE* = 0.22), z (two-tailed) = 4.09, *p* < 0.01 (*p* = 0.004). Despite the differences between the statistics, the average differences were small (Mbaseline = 38.53 compared with Mtreatment = 38.94). The results showed a significant trend. This was possible because the differences between the experimental conditions were maintained in each session (like a trend).

## Discussion

The present study was designed to assess whether different seated affordances (on a ball and on a chair) affected the behavioral and cognitive performance of a person with severe acquired brain injury. We hypothesized that the structural coupling of the body and the environment in the ball condition would be better than in the chair condition. The findings indicate that the subject produced a better behavioral and cognitive performance when working on the therapeutic ball than when working on the chair (the traditional postural setting in cognitive rehabilitation).

The results indicate that the woman managed misbehaviours (physical and verbal) better while working on the ball. We believe these better results are because the work on the ball elicits higher automatic body resources than the work on the chair, helping the person avoid irrelevant stimuli from the environment and centring herself in both her body and her task. In addition to the improvement in behavioral management, the woman also significantly improved the cognitive performance variables (matching success and complex task) while working in the ball condition, suggesting that the therapeutic strategy not only has a modulating effect on the externalizing symptoms but also allows better cognitive function. This cognitive outcome is consistent other studies, demonstrating that cognitive function improves when postural control becomes more difficult (Caldwell et al., 2003; Elliott et al., 2005; Barra et al., 2015).

By contrast with our initial premise, both the matching failure and self-verbalization variables behaved differently from our predictions. These results are consistent with some studies that observed that the increase in the exigency of postural control caused a decrease in the success of tasks (Andersson et al., 1998; Vuillerme et al., 2000; Brauer et al., 2001; Riley et al., 2003; Barra et al., 2006; Rapp et al., 2006; Simoneau et al., 2008).

Although the most important evidence in behavioral disorders arise from single-case experimental designs (Alderman et al., 1999; Alderman, 2002; Barlow et al., 2009), which avoid the uncontrolled variance produced by the heterogeneous nature of this disorder (Alderman and Wood, 2013), we find two main limitation in this study. The first one is related to a possible learning effect. The experimental design developed consisted of the application of two seated affordances during 17 sessions, which might produce this undesirable learning effect. Nevertheless, and even if the outcome was affected by learning effect, it would not impact the main outcome of this research because in each session the person performed both seated affordances. The second limitation of this study is related to ecological approach. The development of this study was applied in a therapeutic context, which means it was performed in a place without any noise, disruption, or distortion of social environment. As a future improvement of the proposed study, we suggest to carry out this research in a more ecological environment in order to evaluate how the relation between the seated affordances and the environmental adaptation modulates cognition and behavior in a rehabilitation center, a day center for people with disabilities and the family’s house.

In recent decades, physical therapies have incorporated dual-task training into motor development theory. This intervention requires that an individual maintain balance while simultaneously performing another task (cognitive or motor). There may be some confusion regarding the similarity between dual-task training and the therapeutic intervention that we present; however, the two interventions are not, in fact, similar. We resolve these differences with two arguments. The first argument is that dual-task training is categorized as a therapy based on information processing theory, which is known as computational therapy (Martínez-Pernía et al., in press), whereas our proposal is based on the enactive approach. The second argument is that the majority of dual-task training focuses on motor development; however, the aim of the present study is the improvement of behavioral and cognitive performance. To our knowledge, only three other studies apply a dual-task paradigm to improve cognitive function (Caldwell et al., 2003; Elliott et al., 2005; Barra et al., 2015), and this work is the only study that is based on the enactive paradigm.

### The Particularities of the Enactive Approach in Neurorehabilitation

In recent decades, new theories have arisen in the cognitive sciences to address the study of cognition in an innovative manner. These perspectives may be summaries from four primary perspectives, or in Gallagher’s words, “the 4e approaches of the mind” (Rowlands, 2010): embodied, embedded, enacted, and extended. Despite differences among these perspectives, all have one similar characteristic. These perspectives assign major significance to extra-neuronal structures for the study of cognition. Their theories emphasize the importance of the body and the environment in the emergence of cognition. Currently, these theoretical stances are a primary line of research in non-Cartesian cognitive sciences (Rowlands, 2010): such as philosophy, (Gallagher, 1986, 1995, 2000, 2005; Johnson, 1987; Gallagher and Zahavi, 2008; Shapiro, 2011), neuroscience (Varela et al., 1991; Damasio, 1994, 2003; Thompson and Varela, 2001; Edelman, 2004), psychology (De Jaegher, 2013; McGann et al., 2013), education (van der Schyff, 2015; Lozada and Carro, 2016), and artificial intelligence (Clark, 1998). And although these types of studies remain scarce in the rehabilitation sciences, there are some publications based on this theory, such as studies regarding the rehabilitation of persons who suffer from an experiential disorder called hemiphobia (Martínez-Pernía and Ceric, 2011) and embodied-enactive clinical reasoning in physical therapy (Øberg et al., 2015).

This work may be relevant in the clinical field. This statement is not only based on the fact that our study shows hints of an effective treatment in rehabilitation of persons with severe behavioral and cognitive disorder, but also because it allows people who are usually excluded from conventional therapies [due to the lack of insight and motivation (Burgess and Wood, 1990; Sazbon and Groswasser, 1991) and also because of the severity of their behavioral disorder (Wood, 1987)] to receive a rehabilitation treatment. Traditional rehabilitation programes can only be employed with patients with less sequelae (Wood and Worthington, 2001). As a consequence, the spectrum of more serious impairments falls outside of any possible treatment. From the point of view of neurorehabilitation based on enactive approach, the main problem of classical therapeutic interventions is that they focus on increasing behavioral management by techniques of self-control. These types of strategies can be successful in people with high cognitive levels because such people are able to understand internal and external instructions. Nevertheless, these strategies are not effective in persons with low cognitive levels because such persons are not aware of what is going on internally and what the therapist is demanding (Alderman, 2003). By contrast, the therapeutic strategy based on the enactive approach overcomes this drawback because enactive therapy is working on unconscious structures of the mind; the interaction between the body and the environment modify behavior and cognition without the necessity of self-awareness.

Surprisingly in cognitive therapy, the discussion of the importance of the body and the environment has been absented despite the fact that it is a basic element into the therapeutic setting. The reason for this lack of interest is due to the fact that cognitive neurorehabilitation is based on functionalism (Martínez-Pernía et al., in press). From the functionalist perspective, the body is reduced to somatosensory cortex or it is restricted to perceive the stimulus of the environment for being later used by the cognition (Gallagher, 1995, 2005). From this view the body is reduced to provide the “raw sensory input” to the brain, but it does not have any contribution in cognition (Gallagher, 2005). This is the reason why most of clinical interventions do not consider what is the best corporal posture to improve cognitive impairment. As a consequence of this omission, therapy implicitly accepts that the gold standard for this issue is to develop cognitive intervention with the patient seated on a chair, posture that has to be maintained along all session. Although this is the ordinary corporal posture to recover from cognitive impairment there are some therapeutic strategies that assume the corporal work in a more innovative way. For instance, the cognitive diagnosis based on dual-task paradigm and some strategies of the unilateral neglect integrate a corporal therapy more pragmatically than to be seated on a chair. Nevertheless, and in spite of these scarce therapeutic interventions, the theoretical assumptions of cognitive neurorehabilitation do not show interest in knowing how the body and the environment improve the cognitive function. By contrast to the functionalist view, the embodied cognition approaches raise the importance of the body and the environment in the emergence of consciousness. The sensorimotor schemas and the environment are the substrate from which the cognition emerges and from where the perception, attention, memory, thought, reasoning, and so on are shaped. In this way, the structural coupling between the body and the environment provides specific conditions that shape the cognition. From this view cognition can be understood as a dynamic process that is situated prior to brain activity (Gallagher, 1995, 2005; Gallagher and Zahavi, 2008). The body (through its movements and its corporal posture) and the particular characteristics of the environment [that affords the action of specific sensoriomotor programs (e.g., walking, sitting, swimming)] work together to shape the cognition (Gallagher, 2005). The meaning of this theory applied to the field of cognitive neurorehabilitation is that any therapeutic strategy has to consider what is the best interaction process between the body and the environment to favor the recovery of a person from his/her cognitive impairment.

## Conclusion

The enactive paradigm applied in the rehabilitation of a woman in this case study introduces a level of treatment that precedes behavior and cognition and emerges from the relation between seated affordance and environmental adaptation. This theoretical consideration allowed the discovery of a better seated affordance for the improvement behavioral and cognitive performance in our case study.

## Author Contributions

DM-P: Person who developed the original idea, design, statistic, therapist, writer, revision final paper. DH: Design, statistic, writer, revision final paper. DH-A and RC: Writer, revision final paper. SG and MB: Therapist, revision final paper.

## Conflict of Interest Statement

The authors declare that the research was conducted in the absence of any commercial or financial relationships that could be construed as a potential conflict of interest.
